# Antimicrobial resistance patterns of *Acinetobacter baumannii* and *Klebsiella pneumoniae* isolated from dogs presented at a veterinary academic hospital in South Africa

**DOI:** 10.14202/vetworld.2023.1880-1888

**Published:** 2023-09-17

**Authors:** Dikeledi C. Sebola, James W. Oguttu, Marleen M. Kock, Daniel N. Qekwana

**Affiliations:** 1 Section Veterinary Public Health, Department of Paraclinical Sciences, Faculty of Veterinary Science, University of Pretoria, Pretoria, South Africa; 2 Department of Agriculture and Animal Health, College of Agriculture and Environmental Sciences, University of South Africa, Johannesburg, South Africa; 3 Department of Medical Microbiology, University of Pretoria, Pretoria, South Africa; 4 Tshwane Academic Division, National Health Laboratory Service, Pretoria, South Africa

**Keywords:** *Acinetobacter baumannii*, antimicrobial resistance, dogs, ESKAPE, *Klebsiella pneumoniae*, multidrug resistance, veterinary hospital

## Abstract

**Background:**

*Acinetobacter baumannii* and *Klebsiella pneumoniae* are opportunistic bacterial pathogens responsible for hospital-acquired infections in veterinary medicine. Infection with these bacteria always requires urgent antimicrobial therapy. However, there is no evidence of studies that have investigated the antimicrobial drug resistance profile of these organisms in a veterinary setting in South Africa. This study investigated the antimicrobial resistance (AMR) patterns of *A. baumannii* and *K. pneumoniae* from clinical specimens obtained from dogs presented at a veterinary academic hospital. The findings of this study contribute to an improved understanding of the AMR profile of these bacteria in veterinary medicine.

**Materials and Methods:**

Retrospective data of clinical samples from dogs that were positive for *A. baumannii* and *K. pneumoniae* between 2007 and 2013 were used in this study. The antimicrobial susceptibility of the isolates was determined using the disk diffusion method following the Clinical and Laboratory Standards Institute guidelines. The *A. baumannii* isolates were subjected to a panel of 20 antibiotics, while *K. pneumoniae* isolates were subjected to a panel of 22 antibiotics. Data were analyzed using descriptive statistics and presented using tables and figures.

**Results:**

Twenty (n = 20) *A. baumannii* isolates were isolated from bronchoalveolar lavage, foreign objects, bone, urine, skin, blood, ear, nasal, and oral cavity. Almost all *A. baumannii* (95%, 19/20) isolates were resistant to at least one antibiotic, and 60% (12/20) were multidrug-resistant (MDR). *Klebsiella pneumoniae* (n = 56) was isolated from urine, foreign objects, abscesses, ears, eyes, tracheal aspirations, bronchoalveolar lavages, eyes, abdominal aspirates, anal glands, bones, and intestinal and lung biopsies. All *K. pneumoniae* (100%, 56/56) isolates were resistant to at least one antibiotic, and 98% (55/56) were MDR.

**Conclusion:**

Both *A. baumannii* and *K. pneumoniae* were isolated in various clinical tissue samples and exhibited a high prevalence of resistance to multiple antibiotics. In addition, these bacteria exhibited a high prevalence of resistance to β-lactam compared to other classes of antibiotics, which is likely to impact treatment options and patient prognosis.

## Introduction

*Acinetobacter baumannii* and *Klebsiella pneumoniae* belong to the group of bacteria termed “ESKAPE” pathogens, and they are responsible for outbreaks in clinical settings across the globe [1]. This ESKAPE group of bacteria is known to escape the biocidal action of antimicrobials and is associated with increased mortality and healthcare costs in both human and animal medicine [1]. In addition, these bacterial species are among the pathogens for which urgent antimicrobial therapy is required due to their tendency to exhibit a high prevalence of multidrug resistance (MDR) [1, 2].

*Acinetobacter baumannii* is an opportunistic pathogen that usually affects immunocompromised patients [3]. It is a non-motile, aerobic, oxidase-negative, and non-fermentative coccobacilli Gram-negative bacterium [4]. It is ubiquitous and has been isolated from drinking water, food, and soil [4, 5]. *Acinetobacter baumannii* can survive for long periods on dry surfaces. As a result, surfaces of inanimate objects in hospitals can be a source of infection for patients [4, 6]. In humans, *A. baumannii* has been isolated from clinical infections such as pneumonia, bloodstream infections, skin and soft-tissue infections, urinary tract infections (UTIs), and meningitis, while it has been isolated from UTIs, bloodstream infections, and wound infections in dogs [4, 5]. *Acinetobacter baumannii* associated with hospital-acquired infections is MDR and has a high prevalence of resistance to the β-lactam and cephalosporin groups of antibiotics [4]. The high prevalence of resistance to these groups of antibiotics can be attributed to various reasons, including the natural resistance due to the interplay between the outer membrane that provides protection, active efflux pump systems, and the low expression of small-aperture outer membrane porins. [6]. *Klebsiella pneumoniae* is a facultative, anaerobic Gram-negative bacterium belonging to the *Enterobacteriaceae* family. It is an intestinal commensal; however, it has been reported in gastrointestinal diseases, UTIs, pneumonia, bacteremia, pyogenic liver abscesses, and burn and wound infections in both humans and animals [6, 7]. Together with *Escherichia coli*, these bacteria are among the most prevalent organisms in hospital and community settings [6, 8]. *Klebsiella pneumoniae* is an opportunistic pathogen in young, old, and immunocompromised humans [6]. It is an important cause of hospital-acquired wound infections and UTIs in humans [7]. In animals, the bacterium has been reported to cause clinical mastitis, pneumonia, septicemia, bacteremia, UTIs, and polyarthritis [7]. *Klebsiella pneumoniae* exhibits a high prevalence of resistance to multiple antibiotics [6, 7, 9]. It acquires and disseminates resistant genes, including those encoding for the extended spectrum β-lactamases, resulting in resistance to β-lactam antibiotics, including penicillin, cephalosporins, and the monobactam aztreonam [6, 9], and, therefore, limiting treatment options [8, 9].

In South Africa, studies of ESKAPE pathogens have been well-documented in human medicine [2, 10, 11]. However, studies investigating antimicrobial drug resistance among the ESKAPE group of pathogens in veterinary medicine are limited. This study aimed to investigate the antimicrobial resistance (AMR) patterns of *K. pneumoniae* and *A. baumannii* isolated from clinical samples of dogs presented at a veterinary teaching hospital. The findings of this study will contribute to a better understanding of antibiotic resistance among *K. pneumoniae* and *A. baumannii* isolates of veterinary origin. In addition, it is envisaged that information generated from this study will be used to guide the treatment of *K. pneumoniae* and *A. baumannii* infections and improve treatment outcomes in a veterinary setting [6].

## Materials and Methods

### Ethical approval

Written consent granting the Academic Teaching Hospital permission to use information obtained from dogs presented at the hospital for teaching and research purposes was obtained from the owners of the dogs. In addition, this study followed all ethical standards for research without direct contact with human or animal subjects. Ethical clearance was also obtained from the University of Pretoria’s Faculty of Veterinary Science Research Ethics Committee, Faculty of Humanities Research Ethics Committee (Project number: REC009-21), and Faculty of Health Sciences Research Ethics Committee (Reference No: 187/2022).

### Study period and location

The retrospective data were processed in November 2022 and analyzed from January 2023 to April 2023. This study was conducted at a veterinary academic hospital in Pretoria, South Africa. The hospital provides clinical services for companion, livestock, and wildlife animals. In addition, the hospital serves as a referral center for internal medicine and surgical cases for clients in and around Pretoria. The bacteriology laboratory in the Department of Veterinary Tropical Diseases that cultured the isolates provides a service to the veterinary academic hospital for routine clinical diagnosis of suspected infectious diseases.

### Data source

Retrospective data records of dog clinical samples submitted to the Bacteriology Laboratory from January 2007 to December 2013 were used in the study. For each isolate, the following information was extracted from the paper records: the patient’s unique number, specimen type, date of sample collection, organ system, and antimicrobial susceptibility test results of the isolates. The data were then entered and stored in an electronic database for analysis.

### Bacterial isolates and antimicrobial susceptibility testing

All the submitted clinical samples were cultured to isolate *A. baumannii* and *K. pneumoniae* using standard bacteriological methods described by Ricketts [12]. Antimicrobial susceptibility testing was performed using the disk diffusion method following Clinical Laboratory Standards Institute (CLSI) guidelines (CLSI 2007, 2008, 2009, 2010, 2011, and 2012) to conduct antimicrobial susceptibility testing.

*Acinetobacter baumannii* isolates were subjected to a panel of 20 antibiotics: amikacin (30 μg), ampicillin (10 μg), carbenicillin (100 μg), ceftazidime (30 μg), cephalothin (30 μg), chloramphenicol (30 μg), enrofloxacin (5 μg), gentamicin (10 μg), imipenem (10 μg), kanamycin (30 μg), lincomycin (10 μg), lincomycin-spectinomycin (100 μg), orbifloxacin (5 μg), oxytetracycline (30 μg), penicillin G (10 μg), piperacillin (100 μg), trimethoprim-sulphamethoxazole (25 μg), amoxicillin/clavulanic acid (20/10 μg), tobramycin (10 μg), and tylosin (15 μg) (Oxoid Ltd., Cambridge, UK).

*Klebsiella pneumoniae* isolates were subjected to a panel of 22 antibiotics: amikacin (30 μg), ampicillin (10 μg), carbenicillin (100 μg), ceftazidime (30 μg), cephalothin (30 μg), chloramphenicol (30 μg), enrofloxacin (5 μg), erythromycin (15 μg), gentamicin (10 μg), imipenem (10 μg), kanamycin (30 μg), lincomycin (10 μg), lincomycin-spectinomycin (100 μg), orbifloxacin (5 μg), oxytetracycline (30 μg), penicillin G (10 μg), piperacillin (100 μg), rifampin (30 μg), trimethoprim-sulphamethoxazole (25 μg), amoxicillin/clavulanic acid (20/10 μg), tobramycin (10 μg), and tylosin (15 μg) (Oxoid Ltd.).

The results of antibiograms were classified as intermediate, susceptible, or resistant, following the CLSI guidelines (CLSI, 2007, 2008, 2009, 2010, 2011, and 2012). For the purposes of this study, resistance to at least one antibiotic was classified as AMR. Multidrug resistance was defined as resistance to at least one antibiotic in three or more antibiotic categories [13].

Antimicrobials to which the bacteria have an inherent resistance were excluded from MDR analysis. For example, *K. pneumoniae* is known to be inherently resistant to ampicillin, carbenicillin, and erythromycin. Therefore, these groups of antibiotics were excluded from the MDR analysis. Since *A. baumannii* is inherently resistant to penicillins and lincosamides, these two groups were excluded from the analysis to determine the prevalence of MDR. In addition, antibiotics were excluded from the MDR analysis if all isolates were not tested to determine their susceptibility to these antibiotics. Therefore, imipenem, tobramycin, rifamycin, and ceftazidime were excluded from the analysis to determine MDR isolates for *K. pneumoniae*, and imipenem, tobramycin, and ceftazidime were excluded from the analysis to determine MDR isolates for *A. baumannii*.

### Data management and analysis

The dataset was assessed for duplicates and missing information, such as the lack of antibiogram results. Some isolates had missing information, but there were no duplicates in the dataset. Isolates from specimens such as endotracheal tubes, screws, pins, wires, catheter tips, nails, and plates were classified as “foreign objects,” while specimens such as lung, liver, spleen, lymph node, heart, and kidney were reclassified as “organ pool.” Crude percentages of isolates of *A. baumannii and K. pneumoniae* that were AMR and MDR were computed and presented as figures and tables. All statistical analyses were performed using the statistical analysis system.

## Results

### Acinetobacter baumannii

**Figure-1:**
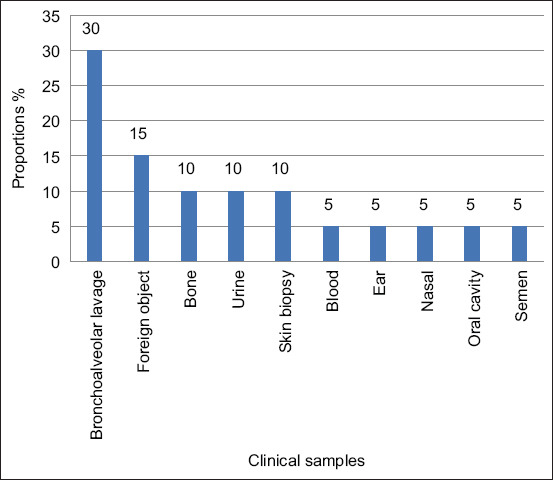
Distribution of *Acinetobacter baumannii* in the various canine samples tested by the bacteriology laboratory at the faculty of veterinary science between 2007 and 2013.

A total of 20 *A. baumannii* were isolated over the study period with six (n = 6; 30%) from bronchoalveolar lavage and three (n = 3; 15%) from foreign objects. *Acinetobacter baumannii* was also isolated from various samples/tissues such as bone, urine, skin, blood, ear, nasal, and oral cavity (Figure-1).

Nineteen isolates were AMR (95%, 19/20) with the majority of *A. baumannii* isolates showing resistance to penicillin G (85%) and ampicillin (65%). Forty-five percentages (45%, 9/20) of the isolates were resistant to amoxicillin/clavulanic acid. All five isolates (100%) tested were resistant to carbenicillin, piperacillin, and ceftazidime. A high prevalence of resistance was recorded against lincomycin (95%), tylosin (68%), chloramphenicol (60%), lincomycin-spectinomycin (60%), and cephalexin (60%). A low prevalence of resistance among the *A. baumannii* was reported for aminoglycosides, except for tobramycin. Similarly, low resistance was observed against fluoroquinolones, tetracycline, and potentiated sulfonamides. One out of four (1/4, 25%) isolates was resistant to imipenem (Table-1).

**Table-1:** Antimicrobial resistance and multidrug resistance profile of *Acinetobacter baumannii* isolated from canine clinical samples tested at a veterinary academic hospital, in South Africa.

Antimicrobial category	Resistant	

	Isolates (n) %	MDR isolates (n) %
Macrolides		
Tylosine	68 (13/19)	-
β-lactams		
Penicillins		
Ampicillin	65 (13/20)	-
Carbenicillin	100 (5/5)	-
Penicillin G	85 (17/20)	-
Piperacillin	100 (5/5)	-
Cephalosporins		
Ceftazidime	100 (5/5)	-
Cephalothin/lexin	60 (12/20)	92 (11/12)
Combination		
Amoxicillin/clavulanic acid	45 (9/20)	75 (9/12)
Carbapenem		
Imipenem	25 (1/4)	-
Aminoglycosides		
Amikacin	30 (6/20)	50 (6/12)
Gentamicin	20 (4/20)	33 (4/12)
Kanamycin	47 (9/19)	75 (9/12)
Tobramycin	80 (4/5)	-
Lincosamides		-
Lincomycin	95 (19/20)	-
Lincomycin-spectinomycin	60 (12/20)	-
Potentiated sulfas		
Trimethoprim-sulphamethoxazole	45 (9/20)	75 (9/12)
Fluoroquinolones		
Orbifloxacin	40 (8/20)	67 (8/12)
Enrofloxacin	45 (9/20)	75 (9/12)
Tetracycline		
Oxytetracycline	35 (7/20)	50 (6/12)
Amphenicols		
Chloramphenicol	60 (9/15)	75 (9/12)

MDR=Multidrug-resistant

Sixty percentages (60%, 12/20) of *A. baumannii* isolates were MDR. A high proportion of isolates exhibited resistance to cephalothin (92%), followed by chloramphenicol, trimethoprim-sulphamethoxazole, enrofloxacin, amoxicillin/clavulanic acid, and kanamycin, to which 75% of the isolates were resistant (Table-1). Three (n = 3) MDR *A. baumannii* isolates were resistant to ten antimicrobials, two (n = 2) to nine antimicrobials, and one (n = 1) to eight antimicrobials (Table-2).

**Table-2:** Antibiotics resistance patterns of *Acinetobacter baumannii* isolated from dog samples presented in a veterinary academic hospital in South Africa.

Patterns	Number
AMI_CEP_CHL_OXY_ENR_GEN_KAN_ORB_SUL_SYN	3
AMI_CEP_CHL_OXY_ENR_KAN_ORB_SUL_SYN	2
CEP_CHL_SYN	2
CEP_CHL_ENR_GEN_KAN_ORB_SUL_SYN	1
CEP_OXY_ENR_ORB_SUL_SYN_	1
CEP_CHL_ENR_KAN_ORB_SUL	1
AMI_CEP_KAN_	1
ENR_KAN_SUL	1
	Total=12

AMI=Amikacin, CEP=Cephalothin/lexin, CHL=Chlorpenicol, OXY=Oxytetracycline, ENR=Enrofloxacin, GEN=Gentamicin, KAN=Kanamycin, ORB=Orbifloxacin, SUL=Trimethoprim-sulphamethoxazole SYN=Synulox

### Klebsiella pneumoniae

**Figure-2:**
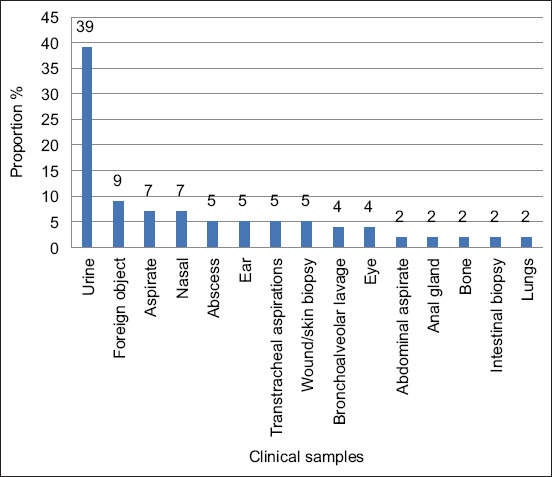
Distribution of Klebsiella pneumoniae in the various canine clinical samples tested by the bacteriology laboratory at the faculty of veterinary science between 2007 and 2013.

A total of 56 *K. pneumoniae* isolates were recorded. Of these, 39% (22/56) were isolated from urine followed by 9% (5/56) from foreign objects. Very low proportions were isolated from abscesses, ears, eyes, transtracheal aspirations, bronchoalveolar lavage, abdominal aspirates, anal glands, bones, intestinal biopsies, and lung biopsies (Figure-2).

All *K. pneumoniae* isolates (56; 100%) were resistant to penicillin G, amoxicillin, carbenicillin, piperacillin, ceftazidime, and lincomycin. Sixty-four percentages (35/56) of the isolates were resistant to cephalexin and 60% to amoxicillin/clavulanic acid. None of the isolates tested were resistant to imipenem**.** *Klebsiella pneumoniae* exhibited a high prevalence of resistance to antibiotics belonging to aminoglycosides, tobramycin (88%), and kanamycin (63%). Ninety-four percentages (94%) of the isolates were resistant to tylosin and 70% to oxytetracycline. One (n = 1) *K. pneumoniae* isolate tested showed resistance to both erythromycin and rifampin. Ninety-eight percentages (98%) of resistant *K. pneumoniae* isolates were MDR, with most being resistant to penicillin G (100%), lincomycin (98%), and tylosin tartrate (93%) (Table-3). The most common resistance pattern among the MDR *K. pneumoniae* isolates included the combination of lincomycin-penicillin G – tylosin (Table-4).

**Table-3:** Antimicrobial resistance and multidrug resistance profile of *Klebsiella pneumoniae* isolated from canine clinical samples tested at a veterinary academic hospital, in South Africa.

Antimicrobial category	Resistant	

	Isolates (n) %	MDR isolates (n) %
Macrolides		
Erythromycin	100 (1/1)	-
Tylosine	94 (51/54)	93 (51/55)
β-lactams		
Penicillins		
Ampicillin	100 (56/56)	-
Carbenicillin	100 (7/7)	-
Penicillin G	100 (56/56)	100 (55/55)
Piperacillin	100 (8/8)	-
Cephalosporins		
Ceftazidime	100 (8/8)	-
Cephalothin/lexin	64 (35/55)	64 (35/55)
Combination		
Amoxicillin/clavulanic acid	60 (33/55)	60 (33/55)
Carbapenem		
Imipenem	0 (0/8)	-
Aminoglycosides		
Amikacin	48 (27/56)	49 (27/55)
Gentamicin	41 (23/56)	42 (23/55)
Kanamycin	63 (33/52)	60 (33/55)
Tobramycin	88 (7/8)	-
Lincosamides		
Lincomycin	100 (54/54)	-
lincomycin-spectinomycin	72 (38/53)	
Rifamycin		
Rifampin	100 (1/1)	-
Potentiated sulfas		
Trimethoprim-sulphamethoxazole	36 (20/56)	36 (20/55)
Fluoroquinolones		
Enrofloxacin	39 (22/56)	38 (21/55)
Orbifloxacin	49 (26/53)	47 (26/55)
Amphenicols		
Chloramphenicol	41 (19/46)	35 (19/55)
Tetracycline		
Oxytetracycline	70 (39/56)	71 (39/55)

MDR=Multidrug-resistant

**Table-4:** Antibiotics resistance patterns MDR-*Klebsiella pneumoniae* isolated from dog samples presented in a veterinary academic hospital in South Africa.

Pattern	Number
LIN_PNG_TYL	7
AMI_CEP_CHL_OXY_ENR_GEN_KAN_LIN_LCS_ORB_PNG_SYN_TYL	3
AMI_CEP_OXY_ENR_GEN_KAN_LIN_LCS_ORB_PNG_SUL_SYN_TYL	2
AMI_CEF_CEP_CHL_OXY_GEN_KAN_LIN_LCS_ORB_PNG_PIP_SUL_SYN_TOB_TYL	2
AMI_CEF_CEP_CHL_OXY_ENR_GEN_KAN_LIN_LCS_ORB_PNG_PIP_SUL_SYN_TOB_TYL	2
AMI_CEP_CHL_OXY_ENR_GEN_KAN_LIN_LCS_ORB_PNG_SUL_SYN_TYL	2
AMI_CEP_OXY_GEN_KAN_LIN_LCS_PNG_SYN_TYL	2
CEP_CHL_OXY_ENR_GEN_KAN_LIN_LCS_ORB_PNG_SUL_SYN_TYL	1
OXY_LIN_LCS_PNG_SUL_TYL	1
LIN_LCS_PNG_TYL	1
AMI_CEP_OXY_ENR_GEN_KAN_LIN_LCS_ORB_PNG_SYN_TYL	1
OXY_LIN_PNG_SYN_TYL	1
AMI_CEP_OXY_ENR_GEN_KAN_LIN_LCS_ORB_PNG_TYL	1
AMI_CEP_CHL_OXY_GEN_KAN_LIN_PNG_SYN_TYL	1
AMI_CEP_OXY_GEN_KAN_LIN_LCS_ORB_PNG	1
AMI_CEP_CHL_OXY_KAN_LIN_LCS_ORB_PNG_SUL_SYN_TYL	1
CEP_CHL_OXY_KAN_LIN_LCS_ORB_PNG_SYN_TYL	1
OXY_LIN_LCS_PNG_TYL	1
CEP_ENR_KAN_LIN_ORB_PNG_SYN_TYL	1
AMI_CEP_OXY_ENR_GEN_KAN_LIN_LCS_ORB_PNG_SUL_SYN_TOB_TYL	1
CEP_OXY_ENR_KAN_LIN_LCS_ORB_PNG_SYN_TYL	1
CEP_CHL_OXY_ENR_LIN_LCS_ORB_PNG_SUL_SYN_TYL	1
CEP_OXY_LIN_LCS_PNG_TYL	1
ENR_LIN_PNG_TYL	1
CEP_KAN_LIN_LCS_PNG_SUL_TYL	1
AMI_CEP_OXY_LIN_LCS_PNG_SUL_TYL	1
CEP_OXY_LIN_LCS_PNG_SUL_SYN_TYL	1
AMI_CEP_PNG_TYL	1
CEP_OXY_LIN_PNG_SYN_TYL	1
AMI_CHL_OXY_ENR_GEN_LIN_PNG_SUL_TYL	1
KAN_LIN_LCS_PNG_SYN_TYL	1
AMI_OXY_KAN_LIN_LCS_PNG_TYL	1
LIN_PNG_SYN	1
AMI_CEF_CEP_CHL_OXY_ENR_GEN_KAN_LIN_LCS_ORB_PNG_PIP_SUL_SYN_TOB_TYL_	1
OXY_KAN_LIN_LCS_PNG_TYL	1
AMI_CEF_CEP_KAN_LIN_LCS_ORB_PNG_PIP_SYN_TYL	1
OXY_LIN_LCS_PNG_SYN_TYL	1
AMI_CEP_CHL_OXY_KAN_LIN_ORB_PNG_SYN_TYL	1
OXY_LIN_PNG	1
AMK_GEN_KAN_LIN_LNC_PNG	1
CEF_CEP_CHL_OXY_ENR_ERT_GEN_KAN_LIN_LCS_ORB_PNG_PIP_RIF_SUL_SYN_TOB_TYL	1
CEF_CEP_CHL_OXY_ENR_KAN_LIN_LCS_ORB_PNG_PIP_SUL_SYN_TOB_TYL	1
Grand Total	55

AMI=Amikacin, CEF=Ceftazidime, CEP=Cephalothin/lexin, CHL=Chloramphenicol, OXY=Oxytetracycline, Enr=Enrofloxacin, ERT=Erythromycin, GEN=Gentamicin, KAN=Kanamycin, LIN=Lincomycin, LCS=Lincospectin, ORB=Orbifloxacin, PNG=Penicillin G, PIP-Piperacillin, RIF=Rifampin, SUL=Trimethoprim-sulphamethoxazole, SYN=Synulox, TOB=Tobramycin, TYL=Tylosine Tartrate, MDR=Multidrug-resistant

## Discussion

This study investigated the AMR patterns of *A. baumannii* and *K. pneumoniae* isolated from dog cases presented at a veterinary hospital in South Africa. Similar to findings reported by other studies, *A. baumannii* and *K. pneumoniae* were isolated from various clinical samples. This confirms past research findings that reported these organisms as associated with various clinical infections in dogs [5, 7, 14–17]. Morever, these organisms have been associated with nosocomial infections and can disseminate resistance genes to other bacteria [18, 19]. Cleaning and disinfection of the environment have proven effective in reducing the burden of these organisms in the environment [20]. However, these organisms can persist in a dry environment and continue to be a source of infection to susceptible patients [14, 20, 21]. Therefore, careful monitoring of dogs admitted to the veterinary hospital through routine surveillance is important to prevent the transmission of these pathogens between patients.

### Antibiotic resistance patterns of *A. baumannii*

Antibiotic resistance among *A. baumannii* isolates is increasing and is associated with increased morbidity, mortality, and treatment costs in the intensive care unit [22]. In this study, a high prevalence of resistance among *A. baumannii* to β-lactam antimicrobials, including penicillin, cephalosporins, and amoxicillin/clavulanic acids, was observed. This is concerning as these antimicrobials are commonly used in small animal practices to treat uncomplicated infections [4]. The high prevalence of resistance observed in this study is consistent with that reported in veterinary studies conducted in the United States of America [23], Switzerland [24], and Malaysia [4]. This is attributed to the wide array of antimicrobial-inactivating enzymes, including β-lactamases, that confer resistance to the β-lactam groups of antimicrobials [18, 24, 25] and the overexpression of the chromosomally encoded AmpC cephalosporinases conferring resistance to broad-spectrum cephalosporins [25, 26].

A low prevalence of resistance to imipenem among *A. baumannii* has been reported in a study by Pailhoriès *et al*. [14]. In this study, only one (1/4) isolate was resistant to imipenem. However, a larger sample size is needed to determine the carbapenem susceptibility profile of *A. baumannii*, considering it is the treatment of choice in humans [5].

*Acinetobacter baumannii* was resistant to trimethoprim-sulphamethoxazole, which is consistent with findings in other studies [27, 28]. This could be due to the overproduction or alteration in plasmid-mediated dihydrofolate reductase associated with trimethoprim resistance [29]. Although *A. baumannii* exhibited resistance to trimethoprim-sulphamethoxazole, evidence suggests that it should be considered for uncomplicated infections [28, 30, 31].

Resistance to aminoglycosides among *A. baumannii* was generally not common in this study, with the exception of tobramycin. This was expected given that resistance to tobramycin among *A. baumannii* increased [32, 33], mainly associated with the synthesis of aminoglycoside-modifying enzymes (AME) and efflux pump systems [32, 34]. This finding has significant public health implications, given that aminoglycosides are commonly used to treat *A. baumannii* infections. Therefore, trends in the susceptibility of these organisms should be monitored [25, 32].

Fluoroquinolones are generally used to treat *A. baumannii* infections in small animals [4]. In this study, a low prevalence of resistance to fluoroquinolones was observed. These organisms’ resistance to fluoroquinolones could be due to the overuse of antibiotics and is mediated by efflux-mediated quinolones resistance [26, 35–37]. Therefore, care is needed to prevent misuse and overuse of fluoroquinolones to curb the development of resistance [38, 39]. A low prevalence of resistance to oxytetracycline was also observed in this study. This is encouraging due to the potential use of tetracyclines as monotherapy or in combination with other antimicrobials for the treatment of *A. baumannii* infections [27, 40, 41].

Forty-five percentages (n = 5; 45%) of *A. baumannii* isolates were MDR. However, a higher prevalence of *A. baumannii* (83.3%, 5/6) from environmental samples exhibiting MDR was reported by Ng *et al*. [4] in a study conducted in Malaysia. The high prevalence of MDR *A. baumannii* is not uncommon [42]. Given this, available evidence suggests that the choices for treatment of MDR *A. baumannii* infections may include carbapenems, colistin, and combination antimicrobials [4, 25, 32, 43].

### Antibiotic resistance patterns of *K. pneumoniae*

Similar to the study conducted in South Korea [20] and France [44], most *K. pneumoniae* isolates in this study were resistant to β-lactam antimicrobials. The β-lactam resistance among *K. pneumoniae* isolates is attributed to the production of the plasmid-mediated sulfhydryl variable-1 a penicillinase [9, 17, 44–46]. However, none of the *K. pneumoniae* isolates in this study exhibited resistance to carbapenems. This is consistent with the findings of Haenni *et al*. [44] in a study conducted in France. These findings suggest that carbapenem could be considered as a treatment option for *K. pneumoniae* [46, 47].

The prevalence of resistance to aminoglycosides varied in this study. For example, low resistance was observed to amikacin and gentamycin [48, 49], while high resistance was observed to tobramycin and kanamycin. The varying prevalence of resistance among aminoglycosides could be attributed to the different resistance mechanisms. For example, resistance to amikacin and gentamicin is associated with the presence of enzymatic modification enzymes (AME) and/or 16S ribosomal RNA methyltransferase (16S-RMTases) [3, 48, 50], whereas tobramycin resistance is associated with the presence of the aminoglycoside N-acetyltransferases (6’)-Ib(-like) protein and not AME or 16S-RMTase genes [51]. Despite the nephrotoxicity of aminoglycosides [52], this group of antimicrobials has been used effectively in the treatment of *K. pneumoniae* infections in both human and veterinary medicine [3].

Consistent with findings from both human and animal studies [16, 17, 46], resistance to enrofloxacin, and orbifloxacin among *K. pneumoniae* isolates was low in this study. Similar to other studies, resistance to trimethoprim-sulphamethoxazole was observed in this study [17, 36]. The low resistance in this study is encouraging, as trimethoprim-sulphamethoxazole is the drug of choice in the treatment of UTIs [17, 53]. In addition, trimethoprim-sulphamethoxazole is effective in the treatment of patients with carbapenemase-producing *K. pneumoniae* infections [54].

Almost all *K. pneumoniae* isolates in this study were MDR. This is not unusual, as AMR genes are frequently observed in this organism [17]. What is of concern is that the role of companion animals as reservoirs for human infections associated with resistant *K. pneumoniae* is not well described in the literature. Therefore, further studies are needed to investigate the transmission of resistant genes between humans and animals.

## Limitation

The data used in this study were limited to one veterinary hospital and excluded other veterinary medical facilities. Since the hospital that provided the data is a referral hospital, it is possible that most isolates may have had previous exposure to antibiotics.

## Conclusion

*Acinetobacter baumannii* and *K. pneumoniae* were identified from various clinical samples suggesting that they are important causes of infections in dogs and can infect various body systems. Both organisms exhibited a high prevalence of resistance to multiple antimicrobials. This has serious veterinary public health implications due to the negative impact on patient treatment and prognosis. Molecular studies are needed to identify genetic drivers of AMR among *A. baumannii* and *K. pneumoniae* organisms. In light of the high prevalence of AMR and MDR observed in this study, the need for strict infection prevention and control measures to prevent the transmission of these organisms in hospital settings cannot be overemphasized.

## Authors’ Contributions

All authors were involved in the study design. DCS: Involved in the data collection and data management, performed all statistical analyses, interpreted the results, and prepared the draft manuscript. DNQ: Involved in data analysis and interpretation of the results, as well as extensive revision of the manuscript. JWO: Extensively reviewed and edited the manuscript. MMK: Involved in the editing of the manuscript. All authors have read, reviewed, and approved the final manuscript.
